# Reducing the Nano-Scale Aggregation of Perylene Diimide Based Acceptor by Conjugating a Bridge with a Large Volume

**DOI:** 10.3390/mi10100640

**Published:** 2019-09-24

**Authors:** Jun-Yi Chen, Xu-Dong Xia, Jicheng Zhang

**Affiliations:** 1 College of Life Sciences, Tarim University, Alar 843300, China; xiaxudong_10@163.com; 2 Department of Chemistry, University of Washington, Seattle, WA 98195, USA; sdzjc2006@163.com

**Keywords:** perylene diimide (PDI), nano-scale aggregation, non-fullerene, polymer solar cells, large volume side chains

## Abstract

A novel perylene diimide (PDI) based acceptor P-PDI was synthesized by attaching a phenyl bridge to two octyloxy side chains. With two large volume side chains, the planarity of P-PDI was significantly reduced, leading to weak nano-aggregation of the PDI groups between the different acceptor molecules. Differential scanning calorimetry (DSC) experiments also revealed that P-PDI was amorphous, and demonstrating the aggregation of P-PDI was successfully suppressed. When blended with PTB7-Th to fabricate a polymer solar cell, a power conversation efficiency (PCE) of 2.21% was achieved, demonstrating that a conjugated bridge with a big volume side chain could significantly reduce the nano-scale aggregation of PDI based acceptor materials, which provides a new strategy to synthesize high efficiency acceptors based on PDI.

## 1. Introduction

Due to its merits of being flexible, light weight, and having low toxicity, polymer solar cells (PSCs) have attracted attention in the last two decades [1,2,3,4]. PSCs usually adopt conjugated polymers as the donor and fullerene derivates such as PC_61_BM or PC_71_BM as the acceptor. Due to the synthesis of novel donor materials and the developing of device fabrication technology, the power conversion efficiency (PCE) has been boosted for single junction PSCs [5]. Although the prospect of PSCs was fascinating, the PCE of PSCs still lagged behind that of inorganic solar cells, which limited their commercial application [6,7]. There are many factors that hamper the increase of PCE in PSCs—one is the weak and narrow absorption of fullerene derivates in the ultraviolet–visible spectroscopy (UV-vis) range, and another one is the difficulty in modifying fullerene based acceptors [8,9,10]. Aside from this, non fullerene organic acceptors have attracted extensive attention. Organic acceptors not only have wide adsorption in the UV-vis range, but they can supply an easy tuning energy level to reduce the energy loss between the donor and acceptor [11,12]. Recently, a PCE of 16% for non fullerene acceptor based PSCs was achieved, demonstrating their potential to break through the limitation of fullerene based acceptors [13]. 

Among the widely investigated non fullerene acceptors, perylene diimide (PDI) based small molecules or polymers have attracted abundant attention because of the merits of high electron mobility [14,15,16]. Although PDI based small molecules as acceptors were intriguing, the strong aggregation caused by the large planar chemical structure of the PDI units usually affects the exciton separation in the blend films [17]. The general way to solve this problem is to introduce a twisty bridge to create a non planar structure, however the over-twisty structure of PDI based small molecules would also induce inferior electron mobility when blended with donor materials [14]. 

In this contribution, a new polymer acceptor based on PDI (P-PDI) was synthesized by the Suzuki reaction of 2,2′-(2,5-Bis(octyloxy)-1,4-phenylene)bis(4,4,5,5-tetramethyl-1,3,2-dioxaborolane) and 5,12-dibromo-2,9-bis(2-ethylhexyl)anthra[2,1,9-def:6,5,10-d′e′f′]diisoquinoline-1,3,8,10(2H,9H)-tetraone (Appendix A.4). By introducing two side chains with a large volume in the phenyl unit, the planarity of the polymer backbones could be reduced and the nano-scale aggregation of PDI between intra molecules was also suppressed because of the big side chains, leading to a more uniform morphology when blended with PTB7-Th. As a result, a PCE of 2.21% with a voltage of 0.82 V was achieved, demonstrating that a conjugated bridge with a big volume side chain could significantly reduce the nano-scale aggregation of PDI based acceptor materials.

## 2. Results and Discussion 

### 2.1. Materials Synthesis and Characterization

**Scheme 1:**
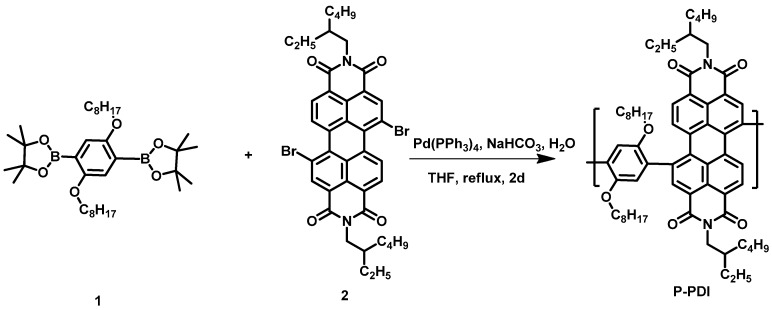
The synthetic route of the polymer acceptor based on perylene diimide (P-PDI).

**Scheme 2:**
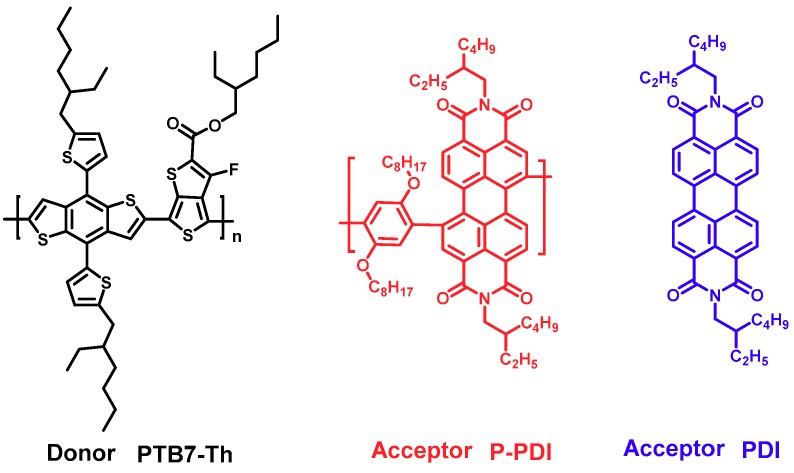
Chemical structure of polymer donor PTB7-Th and acceptors P-PDI and PDI.

As shown in Scheme 1, P-PDI was synthesized by one step of the Suzuki coupling reaction of compound 1 and 2 using tetrahydrofuran (THF) as the solvent, NaHCO_3_ as the base, and Pd(PPh_3_)_4_ as the catalyst procedure. PTB7-Th was also synthesized by the Stille reaction according to the literature [18]. P-PDI can dissolve in common organic solvents such as chloroform (CHCl_3_) and dichlorobenzene (DCB) at room temperature, whereas PTB7-Th could only dissolve in dichlorobenzene (DCB) at an elevated temperature. Measured by gel permeation chromatography (GPC) at a temperature of 150 °C with 1,2,4-trichlorobenzene (TCB) as the eluent, the average molecular weight (*M*_n_) of PTB7-Th and P-PDI was determined to be 47.1 and 66.8 kg/mol, respectively. The chemical structure of polymer donor PTB7-Th, acceptors P-PDI and PDI was shown in Scheme 2. 

### 2.2. Optical Properties

**Figure 1:**
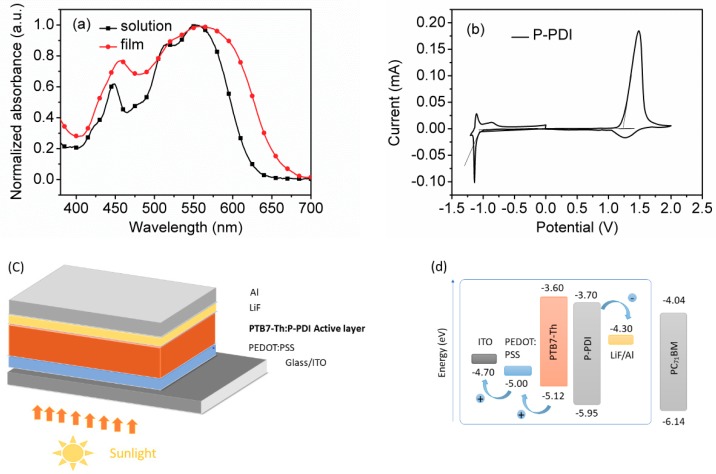
(**a**) Ultraviolet–visible spectroscopy (UV-vis) absorption spectra of P-PDI in solutions and as films; (**b**) cyclic voltammetry (CV) curve of P-PDI; (**c**) Device structure for polymer solar cell (PSC) devices; (**d**) Molecular orbitals of PTB7-Th:P-PDI and PC_71_BM.

To shed light on the light absorption properties of P-PDI, the UV-vis absorption spectra of P-PDI were measured in DCB solutions and as films. As shown in Figure 1a, P-PDI exhibited a broad absorption spectrum in the range from 350 nm to 650 nm in solutions, which might be propitious to generate more photocurrent when blended with PTB7-Th. Upon changing from solutions to films, the absorption peaks were red-shifted, demonstrating that the aggregation in the films was not fully suppressed. The onset adsorption of P-PDI as films is 651 nm, corresponding to the optical band gap (*E*_g,opt_) value of 1.90 eV using the equation: *E*_g,opt_ = 1240/λ_onset._

### 2.3. Electrochemical Properties

To study the electrochemical properties of P-PDI, the cyclic voltammetry (CV) curves of P-PDI films on a glassy carbon working electrode were measured in a CH_3_CN solution (0.1 mol/L Bu_4_NPF_6_). As shown in Figure 1b, the onset oxidation potential of P-PDI was determined to be 1.24 V, and the HOMO energy level of P-PDI was therefore estimated to be −5.95 eV by the equation of *E*_HOMO_ = −e (*E*_ox_ + 4.71 V). Similarly, the reduction potential of P-PDI was measured to be −1.01 V, and the LUMO energy level of P-PDI was therefore estimated to be −3.70 eV according to the same equation. The electrochemical band gap (*E*_g_) was then calculated to be 2.25 eV according to the equation *E*_g_ = *E*_LUMO_ − *E*_HOMO_, which is similar to the value of *E*_g,opt_. As shown in Figure 1d, the LUMO level of P-PDI was slightly higher than that of PC_71_BM, which might lead to higher *V*_oc_ when used as acceptor materials [19,20]. 

### 2.4. Photovoltaic Properties

**Figure 2:**
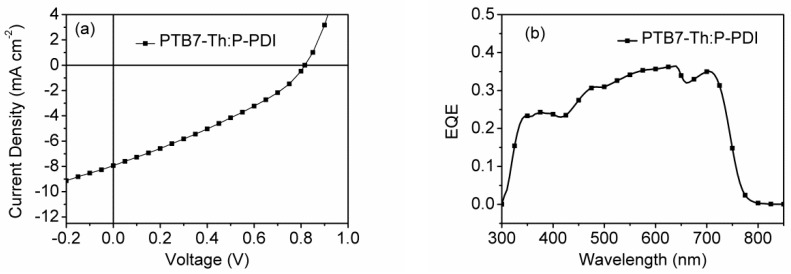
*J-V* (**a**) and external quantum efficiency (EQE) (**b**) curves of PTB7-Th:P-PDI based optimized devices.

**Figure 3:**
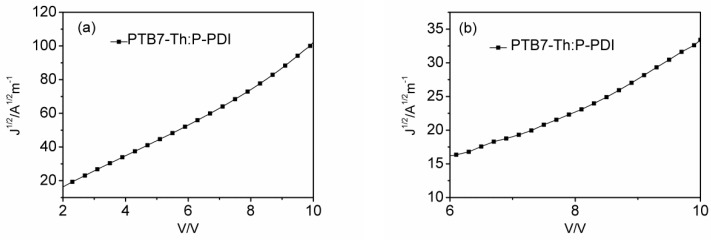
*J*^1/2^-V curves for determining the *μ*_h_ (**a**) and *μ*_e_ (**b**) curves of PTB7-Th:P-PDI based optimized devices.

To investigate the photovoltaic performance of P-PDI as the acceptor in devices, conventional devices with a configuration of ITO/PEDOT:PSS/PTB7-Th:P-PDI/LiF/Al were fabricated, as shown in Figure 1c. DCB was adopted as the solvent to fully dissolve PTB7-Th and P-PDI at 90 °C. The weight ratio of PTB7-Th:P-PDI and the thickness of the blend films was adjusted to optimize the performance of the devices. After optimization, the highest PCE was achieved with a donor/acceptor ratio of 1:2 and a film thickness of 102 nm. The current density voltage (*J-V*) curve of the optimized devices is displayed in Figure 3a and detail parameters are summarized in Table 1. As shown in Figure 2a and Table 1, a PCE of 2.21% was achieved with a *V*_oc_ of 0.82 V, a *J*_sc_ of 7.92 mA/cm^2^, and an fill factor (FF) of 0.34, revealing the potential of P-PDI to be used as acceptor materials. The *V*_oc_ of P-PDI was slightly higher than that based on PC_71_BM, demonstrating that the energy loss between PTB7-Th and acceptors was reduced [19]. The FF of the devices was still not very high, which might be attributed to the unbalanced hole and electron mobilities and the resulting recombination loss in the devices (vide infra) [21]. Besides, the inherent defect of the active layer might also induce some non-geminate recombination like excimers [22], bulk-traps, and surface-traps and may lead to reduced photovoltaic performance [23].

External quantum efficiency (EQE) experiments of PTB7-Th:P-PDI based optimized devices were also carried out to verify the *J*_sc_ of *J-V* test. As shown in Figure 2b, PTB7-Th:P-PDI display a broad response peak in the range from 300 nm to 800 nm with a maximum value of 0.37 at 640 nm, generating more photocurrent in the UV-vis range. The *J*_sc_ value integrated from the EQE curves (7.78 mA cm^−2^) agree well with the *J*_sc_ value from the *J-V* experiments, demonstrating that the *J-V* text results were reliable. 

### 2.5. Transport Properties

To shed light on the carrier transport in the devices, a hole only device with a structure of ITO/PEDOT:PSS/PTB7-Th:P-PDI/Au and an electron only device with a structure of FTO/PTB7-Th:P-PDI/Al were constructed. The hole mobility (*μ*_h_) of PTB7-Th and electron mobility of P-PDI in the devices was therefore determined, respectively, from the dark *J-V* curves of these two devices using the space-charge limited current (SCLC) method. The dark *J-V* curves of these devices were then fitted via the Mott-Guney equation: *J =* 9*ε_o_ε_r_µV*^2^/8*L*^3^, where *ε_r_* is the permittivity of the active layer, *ε_o_* is vacuum permittivity, *µ* is the hole mobility or electron mobility, and *L* is the active layer thickness. As shown in Figure 3, the *μ*_h_ value of the devices with a donor acceptor ratio of 1:2 was calculated to be 4.01 × 10^−5^ cm^2^ V^−1^ s^−1^, whereas the *μ*_e_ value of the devices with a donor acceptor ratio of 1:2 was determined to be 6.41 × 10^−6^ cm^2^ V^−1^ s^−1^, which was two orders of magnitude lower than that of *μ*_h_, resulting in inferior FF. Although the hole mobility and the electron mobility of the devices was not very balanced, the *μ*_e_ of P-PDI was higher than many acceptors [24,25], which could be ascribed to the reduced nano-aggregation of P-PDI. 

### 2.6. Film Morphology

**Figure 4:**
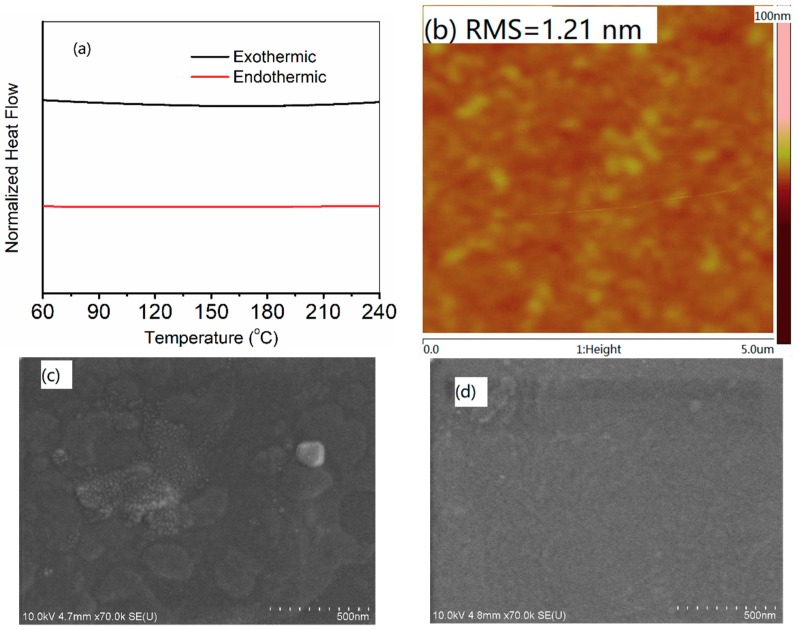

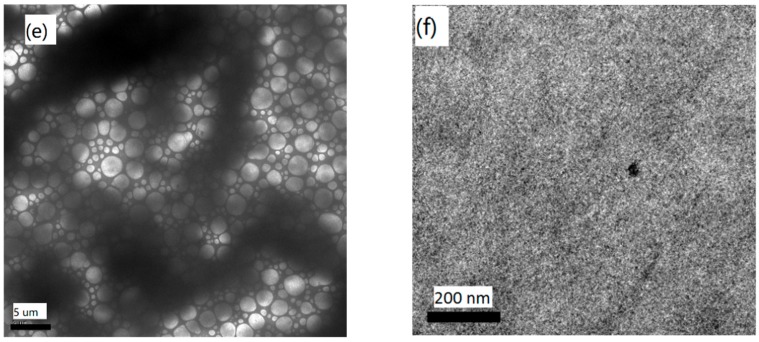
(**a**) DSC curves of P-PDI; (**b**) atomic force microscopy (AFM) height images of PTB7-Th:P-PDI blend films; (**c**) scanning electron microscope (SEM) images of PTB7-Th:PDI blend films (Scale bar: 500 nm); (**d**) SEM images of PTB7-Th:P-PDI blend films (Scale bar: 500 nm); (**e**) transmission electron microscopy (TEM) images of PTB7-Th:PDI blend films (Scale bar: 5 μm) (**f**) TEM images of PTB7-Th:P-PDI blend films (Scale bar: 200 nm).

The main obstacle for PDI based accepters was their strong aggregation as films. When blended with polymer donors, large domains usually formed, leading to inferior exciton separation and low PCE in devices [17,26]. After conjugating PDI with a linker with a large volume side chain, P-PDI displayed no obvious glass transition peak in the range from 60 °C to 240 °C in DSC traces (Figure 4a), revealing that P-PDI was amorphous. To shed light on the film morphology of PTB7-Th:P-PDI blend films, atomic force microscopy (AFM) was used in the tapping mode. As shown in Figure 4a, the blend film displays a pretty smooth film with a root mean square (RMS) roughness of 1.21 nm, demonstrating that no significant aggregates of P-PDI were formed in the blend films [27]. To further investigate the inside film morphology of the active layer, scanning electron microscope (SEM) and transmission electron microscopy (TEM) were also adopted. As shown in Figure 4c–f, the domain size in the PTB7-Th:P-PDI active layer was significantly smaller than PTB7-Th:PDI, revealing that the aggregation of P-PDI was significantly reduced by introducing a bridge with large side groups, and a higher PCE was therefore acquired [28]. 

## 3. Conclusions

In conclusion, a novel PDI based acceptor P-PDI was designed and synthesized. A phenyl unit with two side octyloxy groups was introduced to the polymer backbones to reduce the nano-scale aggregation of the PDI unit. DSC experiments revealed that P-PDI was amorphous, demonstrating that the aggregation of the PDI unit in the acceptors was significantly suppressed. When blended with PTB7-Th to fabricate a non fullerene PSCs, a PCE of 2.21% with a *V*_oc_ of 0.82 V, a *J*_sc_ of 7.92 mA/cm^2^, and an FF of 0.34 was acquired. AFM and TEM of the blend films also displayed rather smooth film morphology, revealing that conjugating a bridge with a large volume could effectively reduce the nano-aggregation of PDI based acceptors. Our results also indicated that P-PDI is a potential acceptor for non fullerene PSCs.

## Figures and Tables

**Table 1:** Summary of the photovoltaic properties of PTB7-Th:P-PDI based devices.

Active Layer	*V*_oc_ (V)	*J*_sc_ (mA·cm^−2^)	*FF*	Power Conversation Efficiency (PCE) (%) Best/Ave ^a^	Film Thickness (nm)
PTB7-Th:P-PDI	0.82	7.92	0.34	2.21/2.10	102

^a^ The average PCE of the devices was achieved from 10 devices.

## Appendix A. Experiment Part

### Appendix A.1. Materials

All chemicals were purchased from commercial sources and used without further purification. Solvents were purified by the standard techniques. 2,2′-(2,5-Bis(octyloxy)-1,4-phenylene)bis(4,4,5,5-tetramethyl-1,3,2-dioxaborolane) (1) and 5,12-dibromo-2,9-bis(2-ethylhexyl)anthra[2,1,9-def:6,5,10-d′e′f′]diisoquinoline-1,3,8,10(2H,9H)-tetraone (2) were synthesized according to the previous literature. Polymer donor PTB7-Th was also achieved by the literature reported [18].

### Appendix A.2. Polymer Solar Cells Fabrication and Characterization

PSCs were fabricated with the device configuration of ITO/PEDOT:PSS (35 nm)/ PTB7-Th:P-PDI/LiF (0.7 nm)/Al (100 nm). The conductivity of ITO is 15 Ω. PEDOT:PSS (Baytron Al 4083 from H.C. Starck) and was filtered with a 0.45 mm polyvinylidenedifluoride (PVDF) film before use. A PEDOT:PSS thin layer was spin-coated on top of the cleaned ITO substrate at 3000 rpm/s for 50 s and was dried at 130 °C for 20 min on a hotplate. A mixture of PTB7-Th and P-PDI in 1,2-dichlorobenzene (DCB) was stirred at 90 °C overnight to ensure sufficient dissolution, and the blend solution was spin-coated onto the substrates with a temperature of 90 °C to form the active layer. The weight concentrations of the blend of PTB7-Th and P-PDI were 25 mg/mL. A top electrode of 0.7 nm LiF and 100 nm of aluminium were thermally evaporated at a pressure of 10^−4^ Pa through a shadow mask. On one substrate, five cells with an effective area of 0.04 cm^2^ each were fabricated. Current-voltage (I-V) and external quantum efficiency (EQE) measurements were conducted in air without encapsulation. The I-V characteristics were recorded at room temperature using an Agilent B2902A Source Meter under the illumination of an AM1.5G AAA class solar simulator (model XES-301S, SAN-EI) with an intensity of 100 mW cm^−2^, and the white light intensity was calibrated with a standard single-crystal Si solar cell.

### Appendix A.3. Space-Charge Limited Current Measurement

Hole-only devices with a structure of ITO/PEDOT:PSS (35 nm)/PTB7-Th:P-PDI/Au (100 nm) and electron-only devices with a configuration of FTO/ PTB7-Th:P-PDI/Al (100 nm) were fabricated. The blend solution of PTB7-Th and P-PDI in DCB was spin-coated onto the PEDOT:PSS layer to form the active layer, like PSC devices, and 100 nm of Au was thermally evaporated at a pressure of 10^−4^ Pa through a shadow mask. For the electron-only devices, the blend solution of PTB7-Th and P-PDI in DCB was spin-coated on the clean FTO substrates to form an active layer. Al electrodes (100 nm) were vacuum-deposited on the polymer thin films. Dark *J–V* curves of the hole-only devices and electron-only devices were measured by the space-charge limited current (SCLC) method.

### Appendix A.4. The Synthesis Route of P-PDI

A mixture of compound 1 (234 mg, 0.40 mmol), compound 2 (309 mg, 0.40 mmol), NaHCO_3_ (0.84 g, 10.0 mmol), THF (10 mL), and H_2_O (2.5 mL) was carefully degassed with N_2_ before and after 5.8 mg Pd(PPh_3_)_4_ was added. The mixture was heated to 90 °C and stirred for 2 days under N_2_ protection. After the reaction, 15.0 mg phenylboronic acid and 0.30 mL bromobenzene were added successively to end cap the end groups. Then, the mixture was poured into 100 mL acetone and collected by filtration. The precipitates were subsequently washed on Soxhlet apparatus by methanol, petroleum ether, and CHCl_3_, respectively.The component dissolved in CHCl_3_ was finally precipitated in methanol and dried under vacuum to afford P-PDI as a dark blue solid (246 mg, 64%). ^1^H NMR (400 MHz, 1,2-dichlorobenzene-d_4_): δ (ppm) 8.93 (m, 2H), 8.78 (m, 2H), 8.36 (m, 2H), 7.98 (m, 2H), 4.30 (m, 4H), 0.85–2.24 (m, 70H). GPC (PS standards): *M*_w_ = 93.1 kg mol^−1^, *M*_n_ = 66.8 kg mol^−1^, and PDI = 1.39.
